# Motivational Tendency Differences Between the Pre-qin Confucianism and Legalism by Psycholinguistic Analysis

**DOI:** 10.3389/fpsyg.2021.724093

**Published:** 2021-11-11

**Authors:** Bo Hu, Miaorong Fan, Feng Huang, Tingshao Zhu

**Affiliations:** ^1^Institute of Psychology, Chinese Academy of Sciences, Beijing, China; ^2^Department of Psychology, University of Chinese Academy of Sciences, Beijing, China

**Keywords:** motivation, Confucianism, Legalism, psycholinguistic, LIWC

## Abstract

Among the hundred schools of thought that flourished during the pre-Qin era, Confucianism and Legalism are the most important ones as their thoughts cast a longstanding influence on the Chinese culture—cultural-psychological formation of the Chinese people. Most of the previous researches focused on analyzing the similarities and differences of the thoughts of Confucianism and Legalism, and few of them analyzed their motivational tendencies. This paper conducted a word frequency analysis of pre-Qin Confucian and Legalist classics with CC-LIWC, an independently developed program for classical text analysis, and made comparative research into the motivational tendencies of the two schools of thought in terms of psycholinguistic differentials. According to our research results, the use of words representing *power* (*M* = 0.1377, *SD* = 0.0104, *p* = 0.014) and *reward* (*M* = 0.0151, *SD* = 0.0042, *p* = 0.037) is more frequent in Legalist classics than in Confucian classics, whereas the use of words representing *affiliation* (*p* = 0.066), *risk* (*p* = 0.086), and *achieve* (*p* = 0.27) shows no significant difference between Confucian and Legalist classics. This paper believes that both Confucianism and Legalism are mainly motivated by power, which is the most distinct feature of their motivational tendencies, and that Legalism is more motivated by power and reward than Confucianism; both Confucianism and Legalism are outcomes of the monarchy society with the former showing the reserved side of monarchy and the latter showing the uninhibited side of monarchy; an effective political methodology is absent in Confucianism, while utilitarianism constitutes the cornerstone of the political philosophy of Legalism.

## Introduction

Confucianism and Legalism both came into being during the pre-Qin period.^1^ Originated in the Spring and Autumn and Warring States period (770–221 BC), Confucianism emphasizes caring about others with benevolence (Zhao, 2011) and makes much of relationships between a monarch and his subjects and between a father and his children (Liu and Li, 2014). Confucianism traces its root back to ancient historical materials and legends and takes spreading the virtues of ancient emperors as its main mission. Confucianism advocates governing a state with ethics (Ren, 2008), upholds “Ren,” “Yi,” “Li,” “Zhi,” and other moral principles (Liu, 2015), and emphasizes mutual rights and obligations between a monarch and his subjects (Chen and Ma, 2018). Representing the ethics of the Chinese agricultural civilization (Ren et al., 2013), Confucianism works to establish a social governance structure based on blood ties and patriarchal relations and pursues benevolent governance by promoting “rules of propriety between ruler and subject, father and son, husband and wife, and elders and juniors,” which is the core political goal of Confucianism.

Severe social upheaval during the Warring States period (475–221 BC) is the seedbed of Legalism. Motivated by the goal to realize political “order,” Legalists inherited the approach of harsh punishments from the Shang Dynasty (c. 1600–1046 BC) and combined “laws + methods + authority” to govern a state (Zhang and Chen, 2016), laying a theoretical foundation for strengthening the centralization of authority. It is a common view that Legalists lay vital importance on “power,” and strengthening the power of a monarch is a strategy and prerequisite for Legalists to restore social order (Peng, 2014). Believing human nature is evil, Legalism advocates governing a state with laws (Qiu, 2017) and emphasizes the control of a monarch over his subjects (Tao and Zhu, 2002).

Compared with Taoism, Mohism and other schools of thought, Confucianism and Legalism play a more prominent part in molding social governance in China (Wang, 2019). Some political ideas and national awareness of Confucianism still impact modern China as its care for people’s livelihood, aspiration for secular achievements and status-oriented ethical view are deeply rooted in the Chinese society (Wei G., 2017). In Contrast, Legalism emphasizes the importance of establishing personal authority, advocates for building a set of strict rules for reward and punishment, and promotes the practice of exerting various means to monitor and control subordinates. Its remarkable ideas on leadership, leader’s responsibilities and leader’s relation with subordinates have profound psychological and behavioral effects on Chinese leaders (Ma and Tsui, 2015; Sun et al., 2020). It can be believed that Confucianism plays a large role in shaping the Chinese people’s perceptions of politics, society and ethics, whereas Legalism produces a far-reaching effect on political rules and practices across different dynasties. On a deeper level, thoughts of Confucianism and Legalism have been internalized into Chinese people’s values, attitude toward life and behavior principles even without being noticed. For instance, there are people who stick to their goals with a strong will (as said in *Analects of Confuciu*s, “the commander of the forces of a large state may be carried off, but the will of even a common man cannot be taken from him.”/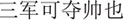,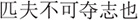); hard workers who believe in painstaking efforts will bring good results (as said in *Mencius*, “when Heaven is about to confer a great responsibility on man, it will first fill his heart with suffering, toil his sinews and bones, exposes his body to hunger.”/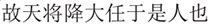,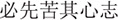,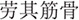,
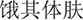
…); intellectuals who uphold moral principles and shoulder responsibilities (as instructed in *Analects of Confuciu*s, “an educated gentleman cannot but be resolute and broad-minded, for he bears great responsibilities and expectations.”/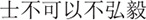,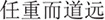); people in power who run a state following Legalist principles (as said in *Han Fei Zi*, “keeping calm and letting things take their own course, you will see flaws.”/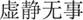,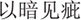).

Many scholars have made comparative studies of Confucianism and Legalism. According to Feng (2013), Confucianism is a type of idealism that values justice over utilitarian benefits while Legalism is a type of realism that uses human nature’s aspiration for utilitarian benefits to develop a series of organizational and leadership skills; Li (2012) laid emphasis on the complementarity between Confucianism and Legalism and analyzed how they were integrated into political practices; Hongbing Song focused on political values and redemptive ideas of the two schools of thought and highlighted how both of them valued creating benefits for the people (Song, 2008); Guan (2013) compared Confucianism and Legalism from the perspective of their origins and confluences. Most of the previous researches focused on analyzing the similarities and differences of the thoughts of Confucianism and Legalism, and few of them analyzed their motivational tendencies. This paper tries to discuss motivational tendencies of Confucianism and Legalism based on quantitative analysis of classical texts, in an effort to provide a new perspective for research in this field.

When it comes to studying traditional Chinese cultural psychology, we can neither conduct a questionnaire-based survey into ancient people’s cultural mentality nor carry out experimental research. Nonetheless, we can apply the psycholinguistics theory and relevant analysis tools to make a quantitative analysis of classical texts. Psycholinguistics is the cross-over study of general psychology and linguistics and takes cognitivism as its theoretical basis (Chen and Li, 2011). Psycholinguistics puts its research focus on the interrelation between linguistic factors and psychological aspects, intending to reveal the psychological processes of a user through linguistic analysis (Wang and Li, 2015). Recently, through psycholinguistics-based quantitative analysis, Li et al. (2019) studied negative responses to live-stream suicides; Su et al. (2020) analyzed the impact of COVID-19 lockdown in Wuhan and Lombardy. From such examples, we can see that psycholinguistics-based quantitative research of psychology is gaining increasing attention.

Motivation is generally conceived as a disposition to strive for a certain kind of satisfaction, such as pride in accomplishment, or the sense of belonging and being warmly received by others, or the feeling of being in control and influential, and as a capacity for satisfaction in the attainment of a certain class of incentives (Atkinson, 1957). Motivation also is believed to be the whole psychological processes that cause, control and maintain physical and psychological activities (Peng, 2012). Many researchers conducted deep studies of the motivation theory, and different researchers have different understandings of the concept and classification of motivation. As for Pennebaker et al. (2015), they used the following five categories of words to describe motivation in the LIWC^2^ dictionary he developed: *affiliation*, *achievement*, *power*, *reward*, and *risk*. We believe that an analysis into the frequency differences of the five categories of words in Confucian classics and Legalism ones can reveal the differences and similarities in motivational tendencies of the two schools of thought to a certain extent. Therefore, this paper focuses its motivational tendencies analysis on these five aspects.

In recent years, text information technology has become a powerful tool for the overall and empirical study of ancient texts (Liu and Li, 2021), and quantitative analysis of texts has drawn increasing attention. Compared with the qualitative approach, the tool-powered quantitative approach could be somewhat more advantageous for psychological studies of traditional Chinese culture as it can deliver text analysis results in a shorter time and researchers do not have to receive training on how to read ancient texts. The Computational Network Psychology Laboratory under the Institute of Psychology, Chinese Academy of Sciences developed CC-LIWC (Classical Chinese-Linguistic Inquiry and Word Count) in 2020 (Fan, 2020). This dictionary is specialized in classical text analysis. Using a huge volume of ancient Chinese texts as a corpus, it applied multiple algorithms to segment words in those texts (Xing and Zhu, 2021). By now, CC-LIWC has proved to be an effective quantitative analysis tool for the research on the psycho-linguistic changes associated with historical celebrities in Henan (Zhao et al., 2021), among others.

Therefore, this paper also chose CC-LIWC for data analysis. To be more specific, this paper used it to compute the use frequency of certain types of words in classical Confucian and Legalist books, so as to analyze differences in their motivational tendencies from the perspective of psycholinguistics. CC-LIWC includes 79 categories of words. Among them, words representing *drives* are a large category, which is further divided into 5 sub-categories: words representing *achievement*, *power*, *reward*, *risk*, and *affiliation*, respectively (same with the word classification method used in LIWC). These 5 sub-categories are the focus of this paper.

## Materials and Methods

### Data Selection

The Confucian and Legalist classics selected by this paper are generally recognized as the best embodiment of the core ideas of the two schools of thought. In terms of Confucian classics, this paper selected 10 from the Thirteen Classics which includes 13 important Confucian classics (Shu, 2011), and they are *Great Learning*, *Doctrine of the Mean*, *Analects of Confucius*, *Mencius*, *Book of History*, *Rites of Zhou*, *Yi Li*, *Zuo Zhuan*, *Gongyang Zhuan*, and *Guliang Zhuan*. As for the other three books included in the Thirteen Classics, *Books of Songs* is a collection of poems and songs, *Book of Changes* is a divination text, and *Erya* is a dictionary. The three books are rather different from the aforementioned 10 books in genre and usage, so they are not covered by this paper. Meanwhile, although Xun Kuang is recognized as a great Confucian philosopher, his thoughts were largely influenced by Legalism (Wang, 2020), making it difficult to label his work *Xunzi* precisely as a Confucian book or a Legalist one. Therefore, this paper did not choose *Xunzi* for research.

Regarding Legalist books, this paper also made a meticulous selection. On the one hand, we considered the works of Guan Zhong, Zichan, and Deng Xi whom many scholars view as Legalism pioneers. Zichan had his state’s code of law cast in a bronze Ding, but he did not have any written ideological works. Deng Xi is not exactly a Legalist as many scholars classify him as a Logician (Gao, 2016). *Guanzi*, named after Guan Zhong, is in fact a synthesis of thoughts of Confucianism, Legalism, Taoism, etc. Nonetheless, seven chapters of *Guanzi* are fundamentally consistent in thoughts with those of Legalists from the states of Wei, Zhao and Han. For this reason, this paper chose the 7 chapters for research, and they are *Fajin*, *Zhongling*, *Fafa*, *Renfa*, *Mingfa*, *Mingfa Jie*, and *Zhengshi* (hereinafter referred to as “*Guanzi Extract*”) (Chai, 2017). On the other hand, we considered works of Li Kui, Wu Qi, Shang Yang, Shen Buhai, Shen Dao, and Han Fei, because they are viewed by Taiyan Zhang and many other scholars as representatives in actively promoting Legalism during the Warring States period (475–221 BC) and bringing about the popularity of Legalism at the time (Wu, 2016). *Fajing* written by Li Kui was lost; *Wuzi* written by Wu Qi is a military work that focuses more on military tactics than on ideological thoughts. Given the above, this paper selected *Book of Lord Shang* by Shang Yang, *Shenzi* by Shen Buhai, *Shenzi* by Shen Dao, and *Han Fei Zi* by Han Fei for Legalism research. Though the number of Legalist books is smaller than that of Confucian ones, those selected by this paper are rather representative as they well embody the essential ideas of Legalism.

To sum up, this paper selected 10 Confucian classics and 5 Legalist classics for research (see Table 1). We accessed their TXT versions through reliable sources and made manual checking to ensure accuracy.

**TABLE 1:** Confucian and Legalist classics selected by this paper.

Confucian Classic	Author(s)	Legalist Classic	Author(s)
*Great learning*	Zengzi	*Guanzi extract*	Guan Zhong et al.
*Doctrine of the mean*	Zisi	*Book of lord shang*	Shang Yang
*Book of history*	Confucius	*Shenzi*	Shen Dao
*Rites of zhou*	Unknown	*Shenzi_sbh*	Shen Buhai
*Yi Li*	Unknown	*Han Fei Zi*	Han Fei
*Zuo Zhuan*	Zuo Qiuming		
*Gongyang Zhuan*	Gong Yanggao		
*Guliang Zhuan*	Gu Liangchi		
*Analects of confucius*	Confucius		
*Mencius*	Mencius		

### Classical Chinese-Linguistic Inquiry and Word Count

First developed by Pennebaker et al. (2015) LIWC is an application of natural language processing technology. This program can analyze texts with quantitative methods. By computing the use frequency of certain categories of words in texts, it can help analyze psycholinguistic differentials and even make personality predictions and social judgments (Wu et al., 2012). Afterward, Chin-Lan Huang et al. from Taiwan developed SC-LIWC, a simplified Chinese version of LIWC (Zhang, 2015). CC-LIWC used by this paper was developed based on SC-LIWC.

This paper focuses on five categories of words: words representing *achievement*, *power*, *reward*, *risk*, and *affiliation* respectively. *Achievement* words (such as *You Cheng*/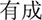 (making achievement), *Zhan Sheng*/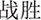 (to triumph) and *Qiu Ming*/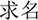 (seeking reputation) in Confucian and/or Legalist classics) are related to striving, failure, aspiration for success, etc.; *power* words [such as *Jun*/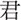
(emperor), *Zhi*/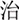 (to govern) and *Guan Jue*/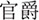 (official ranks and peerage titles)] are connected with social status, social hierarchies, dominance, etc.; *reward* words [such as *Xing Shang*/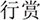 (giving rewards), *Fu*/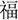
(blessing) and *Yi*/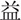
(benefits)] involve rewards, incentives, positive goals, etc.; *risk* words [such as *Shi*/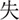 (loss), *Wei*/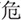
(danger) and *Jin*/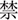
(prohibition)] are in connection with dangers, concerns and things to avoid, etc.; *affiliation* words [such as *Jia*/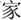 (family), *Qin*/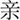
(relative) and *Ai*/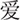 (love)] are those expressing dependence and subordination between people (Pennebaker et al., 2015).

### Data Analysis

**FIGURE 1:**
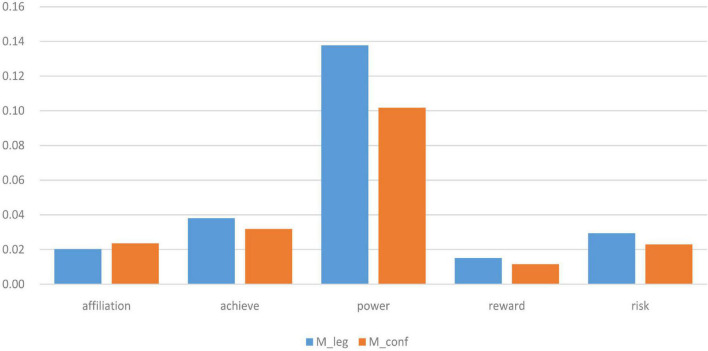
Comparison of average frequencies of each word category in Confucian and Legalist classics. M_leg and M_conf represent the average frequencies of each word category, respectively, in Legalist and Confucian classics.

We took the following four steps to conduct data analysis: first, running the main program of CC-LIWC to compute the total word count (TWC), LIWC word count (LWC), and LIWC cover rate (LCR) of each book (see Table 2); second, calculating the frequency of words standing for *affiliation*, *achievement*, *power*, *reward*, and *risk*, respectively, in each book, and then examining the distribution of the data and calculating the mean, standard deviation (SD), median, and interquartile range (IQR) values of each word category (see Tables 3, 4); third, running a Wilcoxon-Mann-Whitney test^3^ for differences between means of frequencies of each word category in Confucian and Legalist books and calculating the effect size of each category (see Table 5); fourth, using a bar chart to display the average frequency of each word category in Confucian and Legalist books (see Figure 1).

**TABLE 2:** Word counts of Confucian and Legalist classics.

Book	TWC	LWC	LCR
*Guanzi extract*	10,374	8,489	0.8183
*Book of lord shang*	15,888	12,812	0.8063
*Shenzi*	2,753	2,227	0.8089
*Shenzi_sbh*	672	528	0.7857
*Han Fei Zi*	97,338	76,197	0.7828
*Great learning*	1,366	1,109	0.8118
*Doctrine of the mean*	2,773	2,220	0.8005
*Book of history*	19,694	13,642	0.6926
*Rites of zhou*	38,360	26,844	0.6997
*Yi Li*	42,434	29,985	0.7066
*Zuo Zhuan*	15,6211	11,1056	0.7109
*Gongyang Zhuan*	34,277	23,859	0.6961
*Guliang Zhuan*	32,759	22,907	0.6992
*Analects of confucius*	12,085	9,042	0.7482
*Mencius*	27,289	21,035	0.7708

**TABLE 3:** Frequencies of each word category in Confucian and Legalist books.

Category	Books	Affiliation	Achieve	Power	Reward	Risk
Legalist	*Book of lord shang*	0.0218	0.0425	0.1407	0.0152	0.0281
	*Shenzi*	0.0214	0.0283	0.1275	0.0131	0.02
	*Shenzi_sbh*	0.0193	0.0491	0.1399	0.0223	0.0298
	*Guanzi extract*	0.0185	0.0381	0.1525	0.0125	0.0392
	*Han Fei Zi*	0.0201	0.032	0.1279	0.0125	0.0301
Confucian	*Doctrine of the mean*	0.022	0.0346	0.1057	0.0101	0.0238
	*Yi Li*	0.0278	0.04	0.1609	0.0186	0.0148
	*Gongyang Zhuan*	0.0251	0.0176	0.0954	0.0056	0.0213
	*Rites of zhou*	0.0172	0.0535	0.1042	0.0112	0.0218
	*Great learning*	0.03	0.0293	0.0681	0.0168	0.0242
	*Mencius*	0.0235	0.0231	0.0862	0.0111	0.0188
	*Book of history*	0.0191	0.0454	0.1187	0.0101	0.0326
	*Zuo Zhuan*	0.0244	0.0286	0.1088	0.0097	0.0273
	*Analects of confucius*	0.0218	0.0221	0.0744	0.0121	0.0203
	*Guliang Zhuan*	0.0248	0.0245	0.0957	0.0098	0.0239

**TABLE 4:** Statistics of the frequencies of each word category in Confucian and Legalist books.

	Legalist					Confucian				
	Affiliation	Achieve	Power	Reward	Risk	Affiliation	Achieve	Power	Reward	Risk
Mean	0.0203	0.038	**0.1377**	0.0151	0.0294	0.0236	0.0319	**0.1018**	0.0115	0.0229
SD	0.0014	0.0083	0.0104	0.0042	0.0069	0.0038	0.0114	0.026	0.0037	0.0048
Median	0.0201	0.0381	**0.1399**	0.0131	0.0298	0.024	0.0289	**0.0999**	0.0106	0.0228
IQR	0.0021	0.0105	0.0129	0.0026	0.002	0.0032	0.0152	0.0196	0.002	0.0036

**TABLE 5:** Wilcoxon-Mann-Whitney test for differences between means of frequencies of each word category in Confucian and Legalist classics.

Category	*U*-value	*P*-value	M_leg	M_conf	SD_leg	SD_conf	Hedges’g
*affiliation*	–1.8371	0.0662	0.0203	0.0236	0.0014	0.0038	0.765
*achievement*	1.1023	0.2703	0.0380	0.0319	0.0083	0.0114	0.66
*power*	2.4495*	0.0143	0.1377	0.1018	0.0104	0.0260	0.872
*reward*	2.0821*	0.0373	0.0151	0.0115	0.0042	0.0037	0.746
*risk*	1.7146	0.0864	0.0294	0.0229	0.0069	0.0048	0.799

M_leg and M_conf columns show the average frequencies of each word category, respectively, in Legalist and Confucian classics. SD_leg and SD_conf stand for the standard deviations of the frequencies of each word category, respectively, in Legalist and Confucian classics. Hedges’g represents the effect size of each word category. As we can see, the effect size of power words is the largest. *P < 0.05.

## Results

Using the CC-LIWC dictionary, this paper analyzed the frequency differences of words representing *affiliation*, *achievement*, *power*, *reward*, and *risk* used in Confucian and Legalist classics. Table 2 presents word counts and the CC-LIWC cover rate (LCR) of each selected book. Tables 3, 4 list the frequencies of each word category in each book and the data distribution. Table 5 shows the Wilcoxon-Mann-Whitney test for the differences between means of frequencies of each word category in Confucian and Legalist classics. Figure 1 visually presents the differences between means of each word category in Confucian and Legalist classics.

As we can see from Table 2, the highest LCR occurs in *Guanzi Extract* (81.83%) while the smallest LCR occurs in *Book of History* (69.26%). For all the selected books, CC-LIWC realized an LCR of about 75% on average.

Table 3 shows the frequencies of each of the five CC-LIWC word categories in all the selected Confucian and Legalist books. Among the selected books, *Great Learning* has the highest frequency of *affiliation* words (3%); *Rites of Zhou* has the highest frequency of *achievement* words (5.35%); *Yi Li* has the highest frequency of *power* words (16.09%); *Shenzi_sbh* has the highest frequency of *reward* words (2.23%); *Guanzi Extract* has the highest frequency of *risk* words (3.92%).

Table 4 presents the mean, standard deviation (SD), median, and interquartile range (IQR) values of each of the five word categories in the selected Confucian and Legalist classics. Notably, the mean and median values of *power* words (shown in bold in Table 4) rank first in both Confucian and Legalist classics.

As shown in Table 5, the use of words representing *power* (*M* = 0.1377, *SD* = 0.0104, *p* = 0.014) and *reward* (*M* = 0.0151, *SD* = 0.0042, *p* = 0.037) is more frequent in Legalist classics than in Confucian classics, whereas the use of words representing *affiliation* (*p* = 0.066), *risk* (*p* = 0.086) and *achieve* (*p* = 0.27) shows no significant difference between Confucian and Legalist classics.

Figure 1 shows the comparison of average frequencies of each word category respectively in Confucian and Legalist classics. As we can see, the category of *power* words shows the largest difference in average frequencies, and *power* words are much more frequently used than the other four categories.

## Discussion

This paper compares the motivational tendencies of Confucianism and Legalism by computing the frequencies of five categories of words in pre-Qin Confucian and Legalist classics with CC-LIWC. We find that quantitative analysis by psycholinguistic analysis can better and more quickly identify the differences between motivational tendencies of the two schools of thought, a sharp contrast with qualitative analysis which requires huge volume reading of ancient texts. According to our research results, the use of words representing *power* (*M* = 0.1377, *SD* = 0.0104, *p* = 0.014) and *reward* (*M* = 0.0151, *SD* = 0.0042, *p* = 0.037) is more frequent in Legalist classics than in Confucian classics, whereas the use of words representing *affiliation* (*p* = 0.066), *risk* (*p* = 0.086) and *achieve* (*p* = 0.27) shows no significant difference between Confucian and Legalist classics. The category of *power* words shows the largest difference in average frequencies, and *power* words are much more frequently used than the other four categories.

### Royalty Doctrine: The Backbone of Traditional Chinese Thought and Culture

From the perspective of motivational tendencies, both Confucianism and Legalism are much more motivated by power than by any other factors, which may be related to the fact that both of them were subject to monarchy.

China has been a monarchy society ever since it started its recorded history, and overall, royalty doctrine is the backbone of traditional Chinese thought and culture (Liu, 2013). Monarchy supremacy constitutes the core of traditional Chinese political systems (Chen, 2014). Confucianism and Legalism are rooted in the same cultural background—the monarchy society; both of them pursue the goal of “a great order across the land” (Song, 2008) though with different approaches: Confucianism promotes “rules of propriety between ruler and subject and between father and son” whereas Legalism advocates “a hierarchical order between ruler and subject.” Nonetheless, both Confucianism and Legalism have to rely on monarchical powers to realize their political goals, which echoes the highly frequent use of *power* words in both Confucian and Legalist classics.

By comparison, Legalism is more motivated by power than Confucianism. Holding opposite opinions about human nature, Legalists advocate rule by law as they believe in human nature’s longing for material gains and tendency to avoid harmful things (Tao and Zhu, 2002), whereas Confucianists advocate rule by ethics as they believe in moral consciousness (Ma and Li, 2019). Different perspectives on human nature result in different political views, which determines Legalists are more motivated to pursue power while Confucianists are more motivated to create ethical and moral values. The monarch-oriented culture both produced “Ren” and “Li” of Confucianism and harsh punishments of Legalism. The fundamental difference between Confucianism and Legalism lies in that the former represents the reserved side of monarchy while the latter shows the uninhibited side of monarchy. The uninhibited Legalism pursues power more ardently, which probably explains why *power* words are more frequently used in Legalist classics than in Confucian ones.

We believe that Confucianists are more motivated by their pursuit of ethics and morality than by the longing for power and utilitarian benefits. A claim that Confucianism is only motivated to make political accomplishments is disparaging the core values of this school of thought. For Confucianism, “running a state and bringing peace to the world” are just means for achieving “Ren,” meaning virtue, empathy and benevolence. In contrast, Legalists can size up the situation and advance with the times (Wei Z., 2017). Legalists prefer harsh laws and severe punishments over moral education, which may mean they do not recognize the value of moral education and believe in the effective role of power in regulating social order (Tang, 2020). This is a significant ideological difference between the two schools of thought.

### Utilitarianism: The Cornerstone of the Political Philosophy of Legalism

Legalism is much more motivated by reward than Confucianism, which is closely related to the core political concept of Legalism. The skilled use of rewards in governance manifests Legalist deep understanding of human nature.

For Legalists, the aspiration for benefits is the fundamental driving force of individuals (Tao and Zhu, 2002), and it is natural that every man lives for himself since benefits are considered even when people deal with the relationships between ruler and subject and between parents and children (Zhang and Jia, 2018). Such a profound insight of Legalism into human nature is startlingly revealing. Given that, Legalists established sophisticated reward and punishment systems for running the country without any cover-up for their utilitarian pursuit (Shi, 2010). By taking advantage of human nature’s preference for advantages and aversion to disadvantages, Legalists can impel people to do something they don’t want to do, such as giving rewards to encourage people to do farming for war (Li, 1999). This is an important feature of Legalism and can explain why *reward* words are more frequently used in Legalist books than in Confucian ones.

As said by Zhang and Jia (2018) and other researchers, utilitarianism constitutes the cornerstone of the political philosophy of Legalism. Legalism wields both the carrot and the stick. On the one hand, it intensified people’s awareness of rules with punitive measures; on the other hand, it mobilized people to do farming for war by giving rewards. Such a combination of tough and soft tactics proved to be rather effective in a short time. Compared with Legalism’s utilitarian and realistic pursuit, Confucianism’s political ideas are too ideal to be truly realized. Neglecting human nature’s preference for advantages and aversion to disadvantages, Confucian standards for a gentleman are extremely rigorous and almost unreachable. Confucianism upholds “Ren” but fails to develop an effective methodology to act on it, which is a weak spot of Confucianism.

Since the Han Dynasty (BC 206–220), especially since the reign of Emperor Wu who adopted the policy of “respecting Confucianism only,” it seems that Legalism had been abandoned. However, Legalism had been integrated into monarchy tactics and still enjoyed a dominant position (Guan, 2013). Such a combination of Confucianism and Legalism and a blending of force and benevolence are in essence Legalism under a cloak of Confucianism. The goal is to reinforce autocratic monarchy though wrapped in the nice-sounding phrase of “governing the state with ethics” (Sun, 2015). The integration of the two schools of thought during the Han Dynasty seems to be a subjective choice of the monarch, but it is somewhat an inevitable trend to integrate Legalist methodology and Confucian ideology.

It should be noted that a Chinese word may have more than one meaning, which may impact this research to some extent. And the limited number of pre-Qin Confucian and Legalist books results in a small sample size of our research, which may affect the data analysis results to a certain extent. Admittedly, CC-LIWC has some limitations: first, ancient texts tend to be implicit and reserved in semantic expression and can bring about different interpretations from different readers, which poses a challenge to the manual annotation and increases the difficulty of validating the dictionary; second, CC-LIWC has difficulty in determining negative connotations and various rhetorical devices such as metaphor and metonymy used in ancient texts, which may produce false positives of some word categories. In short, there is still room for improving CC-LIWC, which is a focal point of our next work.

There remains great potential for applying psycholinguistics-based quantitative methods to the study of traditional Chinese culture. For instance, we can make a comparative study of different ancient individuals’ self-awareness, positive emotions and negative emotions based on their self-expressive texts (such as recorded dialogues, letters and memorials to the throne); we can also conduct a longitudinal analysis of ancient culture by selecting certain word categories based on certain variables. We believe that the rapid development of computer and big data technologies will bring breakthroughs in the study of traditional Chinese culture and produce new research paradigms. A promising future for the research in this field is unfolding.

## Conclusion

This paper discussed motivational tendencies of different schools of thought based on psycholinguistics. According to the results, both Confucianism and Legalism are mainly motivated by power, which is the most distinct feature of their motivational tendencies, and Legalism is more motivated by power and reward than Confucianism; both Confucianism and Legalism are outcomes of the monarchy society with the former showing the reserved side of monarchy and the latter showing the uninhibited side of monarchy; an effective political methodology is absent in Confucianism, while utilitarianism constitutes the cornerstone of the political philosophy of Legalism.

## Data Availability Statement

The original contributions presented in the study are included in the article/supplementary material, further inquiries can be directed to the corresponding author/s.

## Author Contributions

TZ designed this research. BH wrote the manuscript. MF and FH provided technical guidance for the data analysis. All authors contributed to the article and approved the submitted version.

## Conflict of Interest

The authors declare that the research was conducted in the absence of any commercial or financial relationships that could be construed as a potential conflict of interest.

## Publisher’s Note

All claims expressed in this article are solely those of the authors and do not necessarily represent those of their affiliated organizations, or those of the publisher, the editors and the reviewers. Any product that may be evaluated in this article, or claim that may be made by its manufacturer, is not guaranteed or endorsed by the publisher.

## Footnotes

1 Various schools of thought took shape during the pre-Qin era when their ideas were the purest, so this paper focuses its research on the pre-Qin period. When talking about Confucianists, this paper mainly refers to Confucius, Mencius and their followers during this period; as for Legalists, this paper mainly covers Shen Buhai, Han Fei, and other Legalists from the states of Wei, Zhao, and Han.

2 As an application of natural language processing (NLP), the LIWC (Linguistic Inquiry and Word Count) is a software designed to make word frequency analysis. It mainly comprises a main program and a dictionary. This dictionary can be used to conduct quantitative analysis of designated texts by calculating the frequencies of different categories of words used in the texts.

3 According to the normality test result, the frequencies of all the five categories in Confucian books are not normally distributed, not meeting the requirement for a *t*-test. Therefore, this paper chose to run a Wilcoxon-Mann-Whitney test to examine the differences between means of frequencies of each word category in Confucian and Legalist books.
